# Robust High Mobility Half-Metallic Interface State in CrI_3_/WTe_2_ Based Heterostructures

**DOI:** 10.1021/acsami.5c10764

**Published:** 2025-08-26

**Authors:** Nivedita Pandey, Oscar Grånäs

**Affiliations:** Department of Physics and Astronomy, 99028 Uppsala University, SE-751 20 Uppsala, Sweden

**Keywords:** 1*T*′ and 2H phases, CrO_2_/CrI_3_/WTe_2_/CrO_2_ device, transmission spectrum, temperature-dependent spin-resolved current, tunneling magnetoresistance (TMR), spin filtration efficiency

## Abstract

We report a robust half-metallic interface state in the CrI_3_/2H-WTe_2_ van der Waals (vdW) heterostructure, exhibiting 100% spin polarization and an extraordinary magnetoresistance exceeding 1 × 10^9^%. These unique properties position the CrI_3_/2H-WTe_2_ configuration as an exceptional candidate for applications in data storage, spintronics, and spin caloritronics. By designing a device incorporating CrO_2_ electrodes, we model charge and spin transport in this heterostructure and analyze the thermal properties under parallel (PM) and antiparallel (APM) magnetization states. Using density functional theory (DFT) and nonequilibrium Green’s function (NEGF) methods, we explore how variations in temperature between CrO_2_ electrodes impact spin-polarized currents and demonstrate nearly perfect spin filtration efficiency at low temperatures across both the 1*T*′ and 2H phases of WTe_2_. The device also shows high thermal magnetoresistance (MR), further enhancing its applicability for spin-caloritronic functions. Transmission spectra for PM and APM states reveal temperature-dependent spin transport, reinforcing the heterostructure’s spin filtering effectiveness. This study represents the first detailed investigation of a heterostructure device with CrI_3_ and WTe_2_ phases and CrO_2_ electrodes, highlighting the superior spin filtration and MR capabilities of the CrI_3_/2H-WTe_2_ interface. The robust half-metallic state and remarkable spin filtering efficiency underscore the potential of this structure for developing advanced thermally controlled spintronic and spin-caloritronic devices.

## Graphical abstract

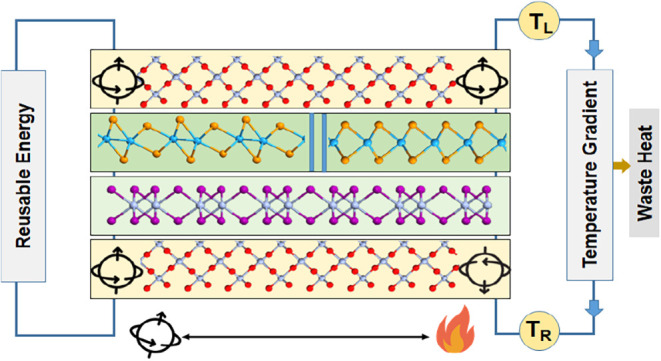

## Introduction

As nanoelectronic devices scale down, dissipation of heat becomes a major challenge, leading to an inefficient use of energy and degraded device performance. Spintronics, an approach leveraging electron spin instead of charge, has emerged as a promising solution for high processing speeds with lower energy consumption. Additionally, thermoelectrics offer a way to convert heat energy into electrical energy, presenting an opportunity for energy recovery in electronic devices.^1–6^ Combining these concepts, the field of spin caloritronics focuses on devices that simultaneously manipulate heat and spin currents, coupling research areas with applications in microelectronics, magnetism, optoelectronics, optospintronics, and thermoelectrics.^7–11^ Key effects in this field include thermal spin filtering, thermal magnetoresistance (TMR), and the spin Seebeck effect.^12–17^

In recent years, advances in two-dimensional (2D) van der Waals (vdW) heterostructures have opened new possibilities for spintronic and spin-caloritronic devices. These layered materials, with weak interlayer bonding, avoid many challenges of conventional heterostructures, such as defects in electrodes, lattice mismatch, and poor thermal stability—all of which traditionally limit TMR. As a result, 2D vdW heterostructures are promising candidates for high-performance spintronic devices like spin filters, spin valves, spin logic circuits, spin tunnel field-effect transistors, and magnetic tunneling junctions (MTJs).^18–22^ Incorporating magnetic layers into these heterostructures creates tunable devices responsive to layer stacking, magnetic fields, and electric fields, thereby affecting the electronic and magnetic properties through modified exchange interactions.^23^

CrI_3_ and its heterostructures are particularly advantageous due to their intrinsic two-dimensional magnetism, stacking dependent magnetism, high spin polarization, and ability to form van der Waals interfaces, making them a good candidate for exploring spin-dependent transport phenomena as demonstrated in systems like CrI_3_/NiCl_2_, h-BN/CrI_3_, Cu/CrI_3_, and VSe_2_/CrI_3_.^24–29^ On the other hand, WTe_2_, a member of the transition metal dichalcogenide (TMD) family, exhibits semimetallic nature, superconductivity, and high magnetoresistance, with applications in energy storage, photovoltaics, and supercapacitors.^30^ WTe_2_ is notable for its multiple polymorphs, with the metallic 1*T*′ phase and semiconducting 2H phase being particularly interesting. Differences in crystal structure between these phases lead to distinct electronic and magnetic properties, making them intriguing candidates for spintronic applications.^31,32^ Despite the promising properties of the 2H phase, it has received limited attention in research.

In this work, we explore the CrI_3_/WTe_2_ heterostructures using the 1*T*′ and 2H phases of WTe_2_. The 2D nature of the CrI_3_/WTe_2_ interface also lends the use of ferromagnetic leads with a high ordering temperature to enhance the critical temperature of the device via the exchange coupling. To this end, we use CrO_2_, a ferromagnetic half-metallic magnet (HMM) electrode. There has been little theoretical work investigating the electronic structure as well as transport properties of heterostructure devices including leads. In particular, for CrI_3_/WTe_2_ heterostructures with CrO_2_ electrodes, we fill this gap by examining both 1*T*′ and 2H phases of WTe_2_. Recent results show that the CrI_3_/1*T*′-WTe_2_ heterostructure is topologically trivial but exhibits a spin-polarized interface state.^21,22^ We show that a similar state is present in the case of CrI_3_/2H-WTe_2_. Moreover, this interface state shows an even more promising behavior in CrI_3_/2H-WTe_2_, exhibiting half-metallic behavior. The 100% spin polarization at the Fermi level enforces maximal spin-selectivity across the device. This unique property significantly enhances the efficiency and functionality of spintronic devices. In devices like spin valves and MTJs, half-metals ensure optimal spin injection and transport, minimizing energy losses and improving performance. The high spin polarization of half-metallic electrodes maximizes TMR, which is critical for applications such as magnetic memory storage and sensing. In the context of spin caloritronics, this implies that the electronic thermal conductivity can become spin-polarized, meaning that heat is predominantly carried by electrons of a particular spin orientation. As a result, the electronic contribution to thermal conductivity can be modulated or even suppressed depending on the relative spin alignment of the ferromagnetic leads.

We use density functional theory (DFT) and quantum ballistic transport calculations based on DFT-derived nonequilibrium Green’s function (NEGF) methods to investigate the electronic, structural, and spin-dependent transport properties of our proposed device configurations, investigating both parallel (PM) and antiparallel (APM) magnetization of the electrodes and assessing their potential for spin-caloritronic applications. The central region of our heterostructure device consists of CrO_2_/CrI_3_/1*T*′-WTe_2_/CrO_2_ or CrO_2_/CrI_3_/2H-WTe_2_/CrO_2_, with CrO_2_ serving as both left and right electrodes, ensuring a smooth potential transition. Open boundary conditions are applied along the transport direction (*z*-axis), and the heterostructure is divided into three main sections for calculations: left electrode, scattering region, and right electrode. Figure 1 shows the device configuration (for the 1*T*′ phase) as well as the structure of the two polymorphs of WTe_2_.

**Figure 1:**
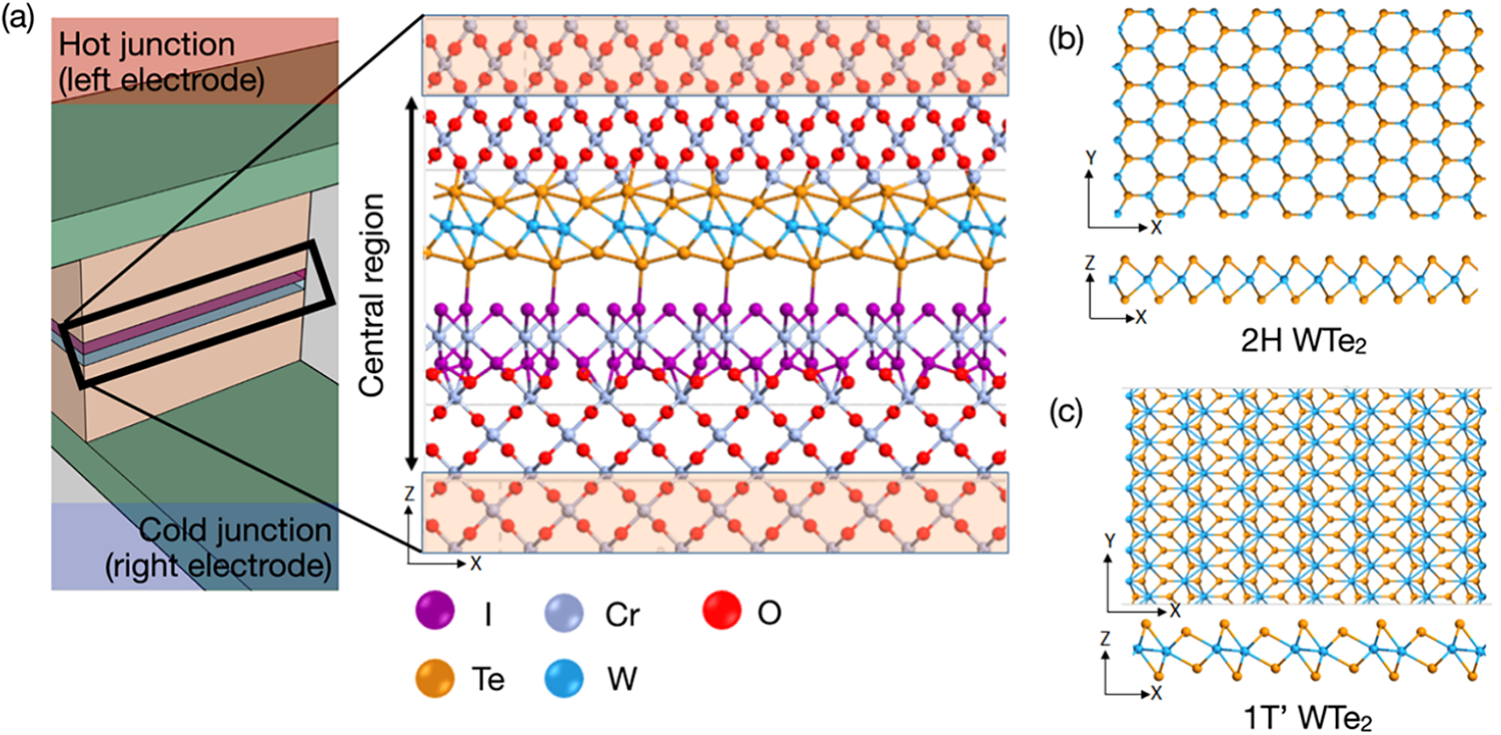
(a) Schematic view of a spin-caloritronic device junction, where the simulated active device region is highlighted. The heterostructure is composed of CrO_2_/CrI_3_/WTe_2_/CrO_2_ using CrO_2_ as the left and right electrodes. Top and side views of (b) 2H-WTe_2_ and (c) 1*T*′-WTe_2_.

## Results and Discussion

Before building the device structure, the structures of the monolayers of CrI_3_, 1*T*′-WTe_2_, 2H-WTe_2_, and the electrode bulk/surface CrO_2_ have been optimized, resulting a lattice constant of *a* = *b* = 6.952 Å, *a* = 3.42 Å, *b* = 6.21 Å, *a* = *b* = 3.12 Å, and *a* = *b* = 4.32 Å, respectively, which is in agreement with the literature.^31,33^ The optimized interatomic distance between the chromium (Cr) and iodine­(I) in CrI_3_ is 2.59 Å, chromium (Cr) and oxygen (O) in CrO_2_ is 1.89 Å, tungsten (W) and tellurium (Te) in 1*T*′-WTe_2_ is 2.73Å, and tungsten (W) and tellurium (Te) in 2H-WTe_2_ is 2.62Å. Further, the heterostructure of CrI_3_ and 1*T*′-WTe_2_ or 2H-WTe_2_ was constructed as 5 × 5 × 1 in-plane supercells. The strain was minimized for each of the constructed heterostructures by first adjusting the optimal supercell dimensions. The electrodes composed of CrO_2_ are further added to both sides of the heterostructure to form a complete vertical device in the *z*-direction. Subsequently, the entire structure is relaxed to find the lowest energy configuration with minimized strain.

In the heterostructure, the CrO_2_ layer experiences 1.8% strain, and the WTe_2_ layer in its 1*T*′ phase undergoes a strain of around 1.2%, while the 2H phase of WTe_2_ shows a strain of 1.9%. The CrI_3_ monolayer exhibits a strain of about 2.4%. These strain values result from lattice matching and structural optimization of the layered materials within the vertical stacking configuration of the heterostructure. The strain values were obtained using a supercell of 5 × 5 × 1 and were minimized using structural optimization via van der Waals corrections. These corrections ensure accurate interlayer spacing and energy minimization, maintaining the material’s intrinsic properties while accommodating the vertical stacking configuration. The small lattice mismatch for the 1*T*′ and 2H phase heterostructures vouches for minor further relaxations and points to the feasibility of experimental fabrication.

In our device, CrO_2_ acts as a half-metallic ferromagnet, CrI_3_ as a ferromagnetic spin filter, and WTe_2_ as a nonmagnetic spacer with potential spin–orbit effects. CrI_3_ maintains a uniform ferromagnetic order in both parallel (PM) and antiparallel (APM) configurations of the CrO_2_ electrodes. There is no magnetic phase transition in CrI_3_ when the electrode alignment changes. Therefore, spin transport is mainly governed by the relative CrO_2_ alignment, with CrI_3_ acting as a stable and effective spin filter. The magnetic configuration of the CrI_3_ layer is crucial in our interpretation of spin-dependent thermal transport. This configuration determines whether the CrI_3_ acts as an efficient thermal spin filter or suppresses the transport.^27,34,35^

Figure 1 shows the full structure repeated in the horizontal plane for better visibility. The source electrode (electrode on the upper left in Figure 1a) has a higher temperature (hot junction), and the drain electrode (electrode on the lower right in Figure 1a) has a lower temperature (cold junction); these are subsequently referred to as left (for hot) and right (for cold) electrodes. After full relaxation, the 1*T*′-based device has a total length of 25.21 Å, while the 2H-based device has a total length of 28.74 Å. The difference in device length can be attributed to the difference in lattice matching and bonding properties between the CrO_2_ layer and the WTe_2_ polymorphs, resulting in longer bond distances in the vertical direction.

To investigate the electronic structure of the full device, we start by computing the ground-state electronic density of states (DOS) for the structure. The calculations are performed for the fully relaxed structures and included spin–orbit coupling. It is known that interlayer coupling may substantially influence the electronic structure. The DOS for the full device is shown in Figure 2, with the 1*T*′ polymorph of WTe_2_ in panel (a) and the 2H polymorph in panel (b). The up-spin channel is plotted with a positive sign, and the down-spin is plotted with a negative sign. As expected, the free-standing monolayer of 1*T*′-WTe_2_ is semimetallic, and the monolayer of the 2H phase of WTe_2_ has a band gap of 0.63 eV when spin–orbit coupling (SOC) is included (0.91 eV without SOC)^36–38^ as shown in Figure 2c,d, respectively. Panels[Fig fig2] (c) and (d) show the partial DOS projected onto the W and Te species of the 1*T*′ and 2H phases, respectively. From the partial DOS, we can see that the 2H phase remains gapped also in the full device structure. Comparing panel (c) to available literature for bilayers of CrI_3_ and 1*T*′-WTe_2_ indicates that a spin-polarized interface state is developed, previously suggested as a candidate topological spin filter.^21^ We note that this state is reproduced also in the device configuration with CrO_2_ leads, indicating a robustness of the reconstructed electronic structure at the interface. More interestingly, the device constructed with a central CrI_3_/2H-WTe_2_ bilayer shows a half-metallic state. The purity of spin and the relatively large spin-up DOS at the Fermi level indicate favorable conditions for highly spin-selective transport properties.

**Figure 2:**
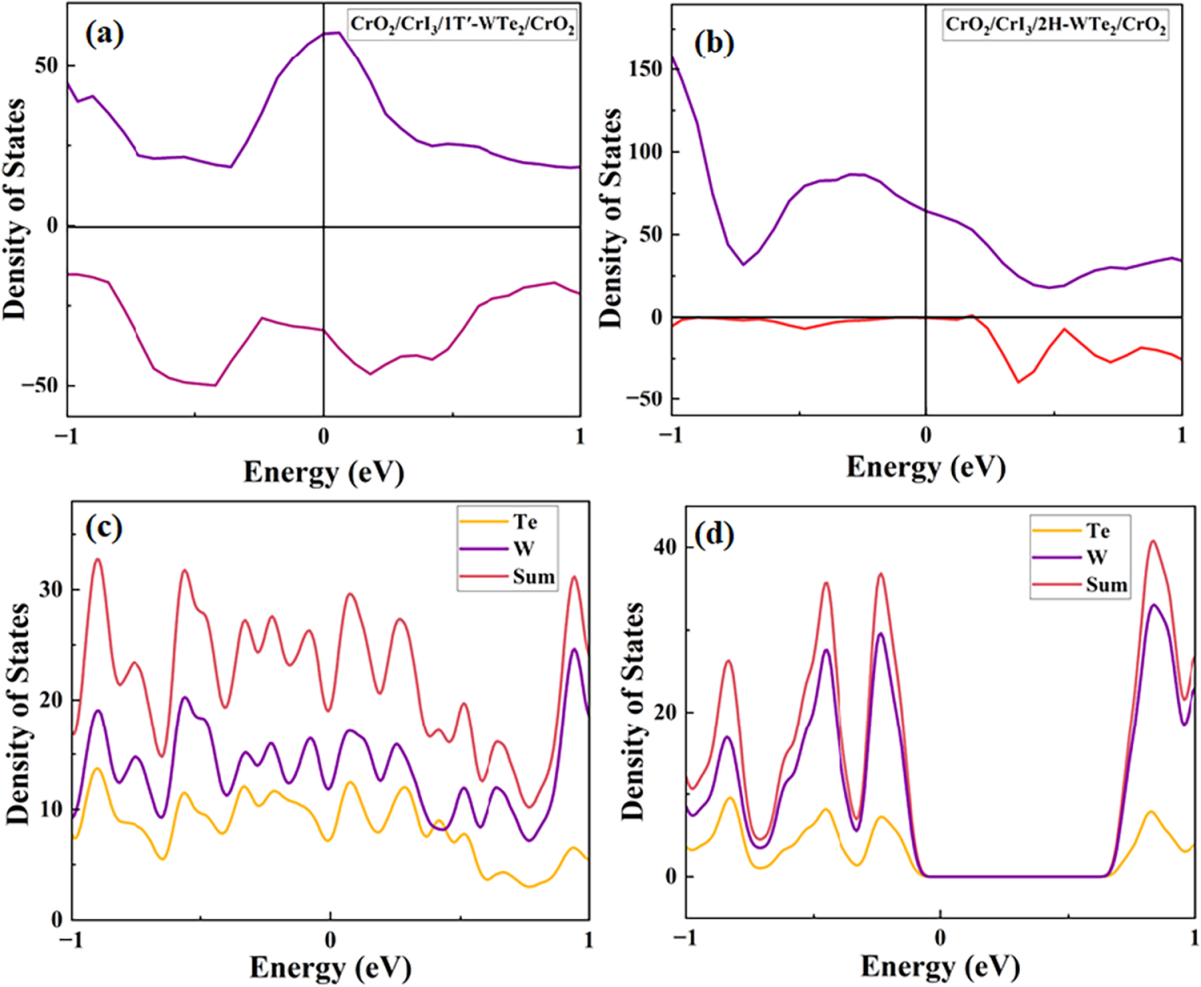
Density of states of (a) a full device based on CrO_2_/CrI_3_/1*T*′-WTe_2_/CrO_2_, (b) a full device based on CrO_2_/CrI_3_/2H-WTe_2_/CrO_2_, (c) the 1*T*′ phase of WTe_2_, and (d) the 2H phase of WTe_2_.

To investigate and understand the spin-selectivity of the devices with respect to a temperature difference between the two electrodes Δ*T*, we examined the transmission function $T^{↑\left(↓\right)}$ of the heterostructure device including both 1*T*′-WTe_2_ and 2H-WTe_2_ phases.^39,40^ The up (down)-spin transmission function determines the probability of electron transfer between the two semi-infinite electrodes and is computed using the NEGF formalism given by the equation:

1 $$T_{\sigma }\left(T\right)=\int_{-\infty }^{\infty }\mathrm{Tr}\left[\Gamma_{\sigma }^{L}\left(E,T\right)G_{\sigma }^{R}\left(E,T\right)\Gamma_{\sigma }^{R}\left(E,T\right)G_{\sigma }^{A}\left(E,T\right)\right]\left(-\frac{\partial f\left(E,\mu ,T\right)}{\partial E}\right)dE,\sigma \in \left\{↑,↓\right\}$$

where *G*_σ_^R^(*E*, *T*) = [(*E* + *i*η)*I* – *H*_eff,σ_(*T*)]^−1^ is the retarded Green’s function, and the advanced Green’s function is its Hermitian conjugate: *G*_σ_^A^(*E*, *T*) = (*G*_σ_^R^(*E*, *T*))^†^. The coupling matrix is given by Γ^L(R)^ = *i*|Σ_L/R_(*E*, *T*) – Σ_L/R_(*E*,*T*)^†^|, and the Fermi–Dirac distribution function is given by $f\left(E,\mu ,T\right)=\frac{1}{1+e^{\left(E-\mu \right)/k_{B}T}}$ , which represents the interchange between the left­(right) electrode, having self-energy as Σ_L/R_ and the scattering region.

Further, computational details can be found in the Methods Section. The calculated spin-dependent transmission function $T^{↑/↓}\left(E\right)$ for both the PM configuration and the APM configuration is shown in Figure 3.

**Figure 3:**
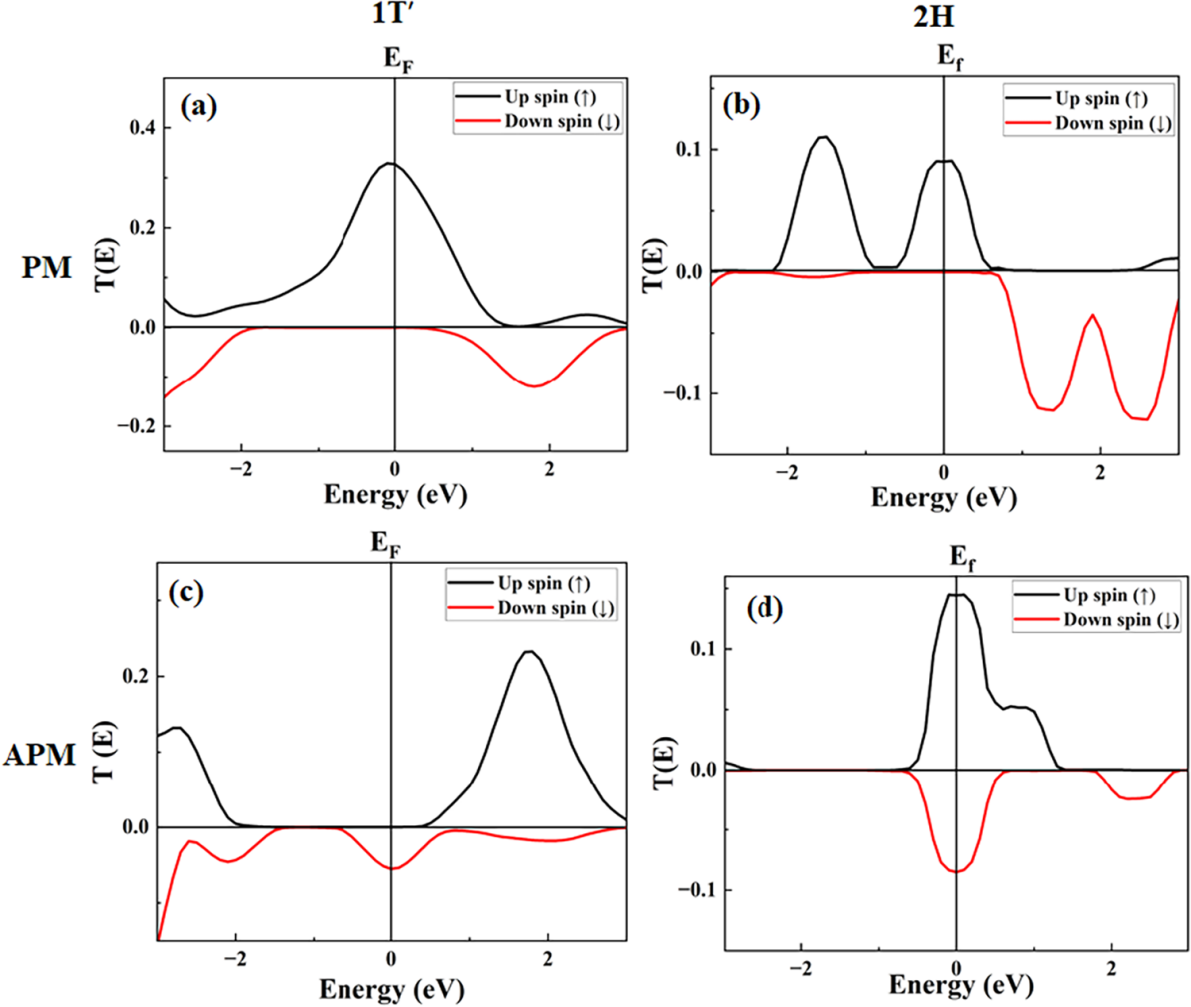
Panels (a) and (c) depict the transmission spectrum curve in the equilibrium for CrO_2_/CrI_3_/1*T*′-WTe_2_/CrO_2_, and panels (b) and (d) depict the transmission spectrum curve in the equilibrium for CrO_2_/CrI_3_/2H-WTe_2_/CrO_2_ for the PM and APM configurations, respectively. The spin-down component is plotted with a negative sign for clarity.

The transmission spectrum will impact the temperature dependence of the spin current due to the asymmetry around the Fermi level. Only a part of the negative and positive currents nullifies each other and produces a net spin current. We have investigated the transmission spectrum of the heterostructure device CrO_2_/CrI_3_/1*T*′-WTe_2_/CrO_2_, where the PM configuration is shown in Figure 3a, and the APM configuration is shown in Figure 3c. For the PM case, the states available for conduction of up-spin electrons are high, in contrast to the down-spin electrons around the Fermi level. It represents that the up-spin electrons are the primary carriers responsible for carrying charges in this case. It also suggests a strong spin filtering effect, where only up-spin electrons contribute to transport. In the APM case, we instead have transmission from the down-spin while the up-spin transmission is nearly zero, which indicates that the charge transport is dominated by spin-down electrons in this state. It further represents complete spin-polarization reversal between the two magnetic configurations, which is a key characteristic of an ideal spin device. Figure 3b,d show the CrO_2_/CrI_3_/2H-WTe_2_/CrO_2_ heterostructure in the PM and APM cases, respectively. For the PM case, as illustrated in Figure 3b, we have transmission of spin-up electrons around the Fermi level, whereas no net hole transmission occurs. It indicates that up-spin electrons are the primary charge carriers, leading to a strong spin polarization in transport. However, for the APM case, both the up- and down-spin electrons are available for conduction, generating a cancellation of the net transmission, as shown in Figure 3d. Also, it suggests partial spin filtering, where up-spin electrons still contribute more to conduction, but down-spin electrons are not entirely blocked. This implies that while the spin polarization of the current is reduced compared to the parallel case, the system still favors up-spin transport.

To determine the spin-dependent current of the junction, computed as a function of electrode temperature, we use the Landauer-Buttiker formula as given below^41^

2 $$I_{↑\left(↓\right)}=\frac{e}{h}\int_{-\infty }^{\infty }T^{↑\left(↓\right)}\left(f_{L}\left(E,T_{L}\right)-f_{R}\left(E,T_{R}\right)\right)dE$$

where *f*_L(R)_(*E*,*T*_L__(*R*)_) is the equilibrium Fermi–Dirac distribution function for the left (right) electrode, which governs the distribution of electrons and the carrier concentration due to the difference in the temperature of the two electrodes. The Landauer-Buttiker formalism allows for a current to be driven purely by the temperature difference between the two electrodes without any bias through the different left and right electrode temperatures appearing in the Fermi–Dirac distribution function. The spin current *I*_sp_ and the charge current *I*_ch_ of the designed two-probe system are given by $I_{\mathrm{sp}}=\frac{\hbar }{2e}\left|I_{↑}-I_{↓}\right|$ and *I*_ch_ = *I*_↑_ + *I*_↓_, respectively.

We have evaluated the spin transport properties of the designed device by studying its thermal spin-dependent current behavior versus the temperature difference (Δ*T* = *T*_L_ – *T*_R_) between left (*T*_L_) and right (*T*_R_) electrodes for PM and APM cases. The spin-dependent current flows due to the difference in temperature between the left (*T*_L_) and right (*T*_R_) electrodes. The up-spin (*I*_↑_) and down-spin (*I*_↓_) components of current have been shown with solid and dashed lines, respectively. For the device CrO_2_/CrI_3_/1*T*′-WTe_2_/CrO_2_, in the PM case (Figure 4a), the up-spin component of the current rises with the (Δ*T*) for *T*_R_ = 20, 40, 60, and 80 K. The down-spin component of the current is entirely filtered out and maintains a zero value in the whole range of Δ*T*. In the APM case (Figure 4c), the trend is opposite to the PM case. The down-spin component of current increases, while the up-spin component is filtered out with the increase in (Δ*T*) for *T*_R_ = 20, 40, 60, and 80 K. Also, the spin-dependent current is 3 orders of magnitude less in the APM case as compared to the PM case. These results also confirm the perfect spin filtering efficiency of the designed heterostructure device. The physical mechanism behind the behavior of the spin-dependent current can be understood from the Landauer-Buttiker formula, as given in eq 2. From eq 2, it is clear that the spin current is dependent on the transmission coefficients and also on the difference of Fermi–Dirac distributions originating in the temperature difference between the left electrode *T*_L_ and right electrode *T*_R_. The difference in the Fermi–Dirac distribution function for the electrodes arise due to the difference in temperature (*T*_L_ > *T*_R_), which will further lead to two different types of carrier: one is due to the carriers having energy higher than the Fermi level, and they move from the hot junction to cold junction leading to generation of electronic current (*I*_e_); the second due to the carriers having lower energy as compare to Fermi level, and the carriers move from cold junction to hot junction causing the generation of hole current (*I*_h_).

**Figure 4:**
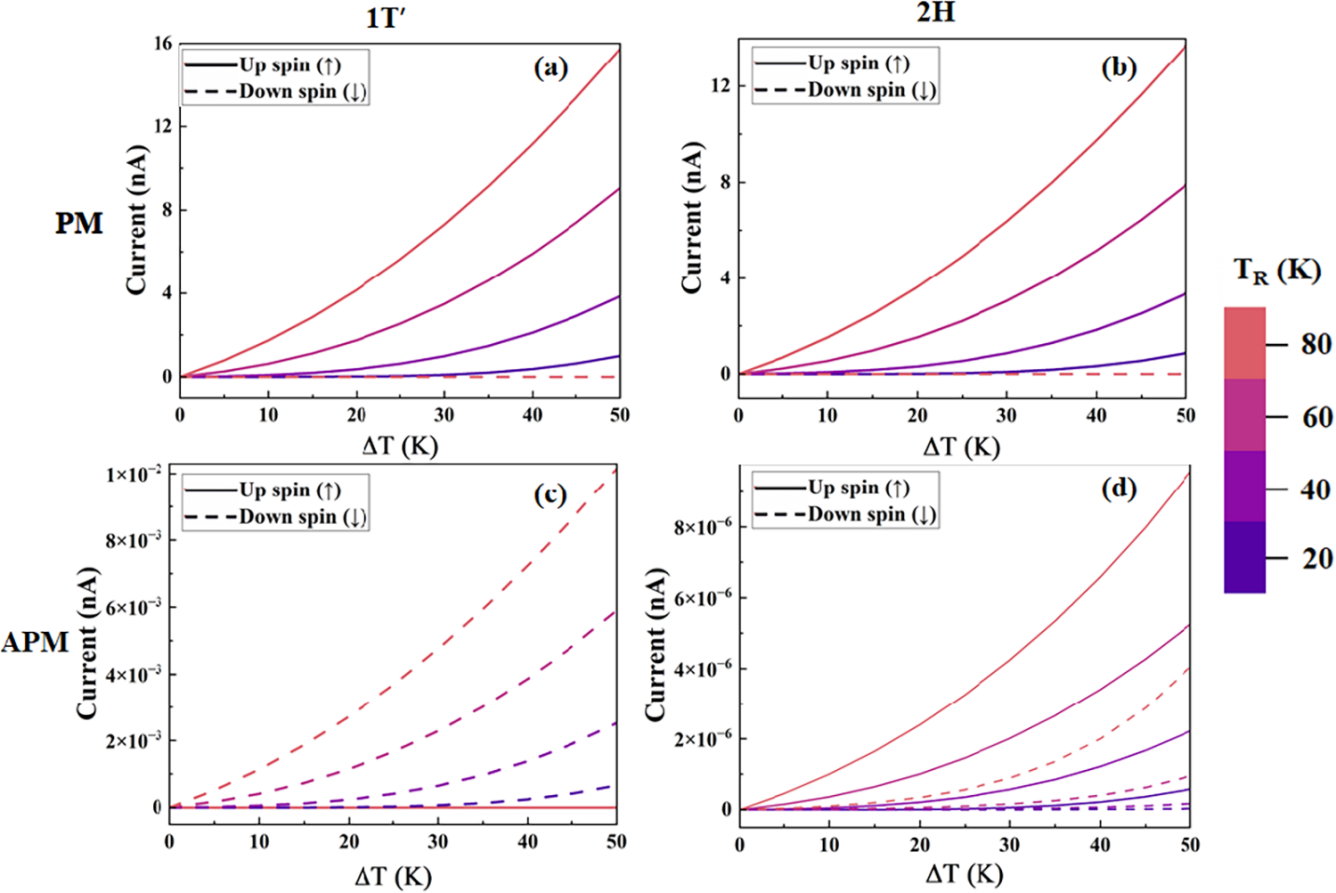
Current per junction for the four cases, consisting of the PM configuration using the 1T’-based (a) and the 2H-based heterostructures (b) and the APM configuration in panels (c) and (d) using the 1T’- and 2H-based heterostructures.

To understand the behavior of temperature-dependent spin current for the designed device CrO_2_/CrI_3_/2H-WTe_2_/CrO_2_ with respect to Δ*T*, we have plotted the results as shown in Figure 4b,d for PM and APM, respectively. For the PM case (Figure 4b), the up-spin component of the current is high compared to the down-spin component of the current. The up-spin component of current rises with an increase in Δ*T* and with an increase in the value of *T*_R_ = 20, 40, 60, and 80 K, while the down-spin current maintains a zero value for all of the computed temperatures. For the APM case (Figure 4d), both up-spin and down-spin components of thermally driven current increase with an increment in Δ*T* and also with an increase in the value of *T*_R_ from 20 to 80 K and are transported through the designed vdW heterostructure device. The current-carrying spin channel in the APM case differs between the 2H phase of WTe_2_, represented in Figure 4d, and the 1*T*′ phase of WTe_2_, represented in Figure 4c. For the 2H phase of WTe_2_, the up-spin channel dominates, with some minor contribution from spin-down, whereas for the APM case of the 1*T*′ phase of WTe_2_ as shown in Figure 4c, the up-spin channel is completely unavailable and only the down-spin component of the current is present. In both cases, the currents are small, but the magnitude is 3 orders of magnitude lower in the APM case for the 2H phase of WTe_2_ as compared to the 1*T*′ phase of WTe_2_.

Further, we have calculated the spin filtration efficiency (η), which can give information about the degree of spin polarization of the temperature-dependent transport current, which is given by

3 $$\eta =\frac{\left(|I_{↑}-I_{↓}|\right)}{\left(|I_{↑}+I_{↓}|\right)}\times 100\%$$

The spin filtration efficiency with respect to Δ*T* for *T*_R_ = 20, 40, 60, and 80 K for the PM and APM cases for CrO_2_/CrI_3_/1T’-WTe_2_/CrO_2_ is shown in the Supporting Information, in which the device showed a perfect 100% spin filtration efficiency for both the PM and APM cases. Figure 5 depicts the spin filtration efficiency with respect to Δ*T* for *T*_R_ = 20, 40, 60, and 80 K for CrO_2_/CrI_3_/2H-WTe_2_/CrO_2_ for the PM and APM cases as shown in Figure 5 a,b, respectively. For the PM case (Figure 5a), the designed heterostructure device CrO_2_/CrI_3_/2H-WTe_2_/CrO_2_ sustains 100% efficiency for all values of *T*_R_, but for the APM case (Figure 5b), a highest efficiency around 92% has been achieved for *T*_R_ = 20 K, and it maintains the value with the increase in Δ*T* value. For *T*_R_ = 40, 60, and 80 K, the spin filtration efficiency decreases with an increase in Δ*T*.

**Figure 5:**
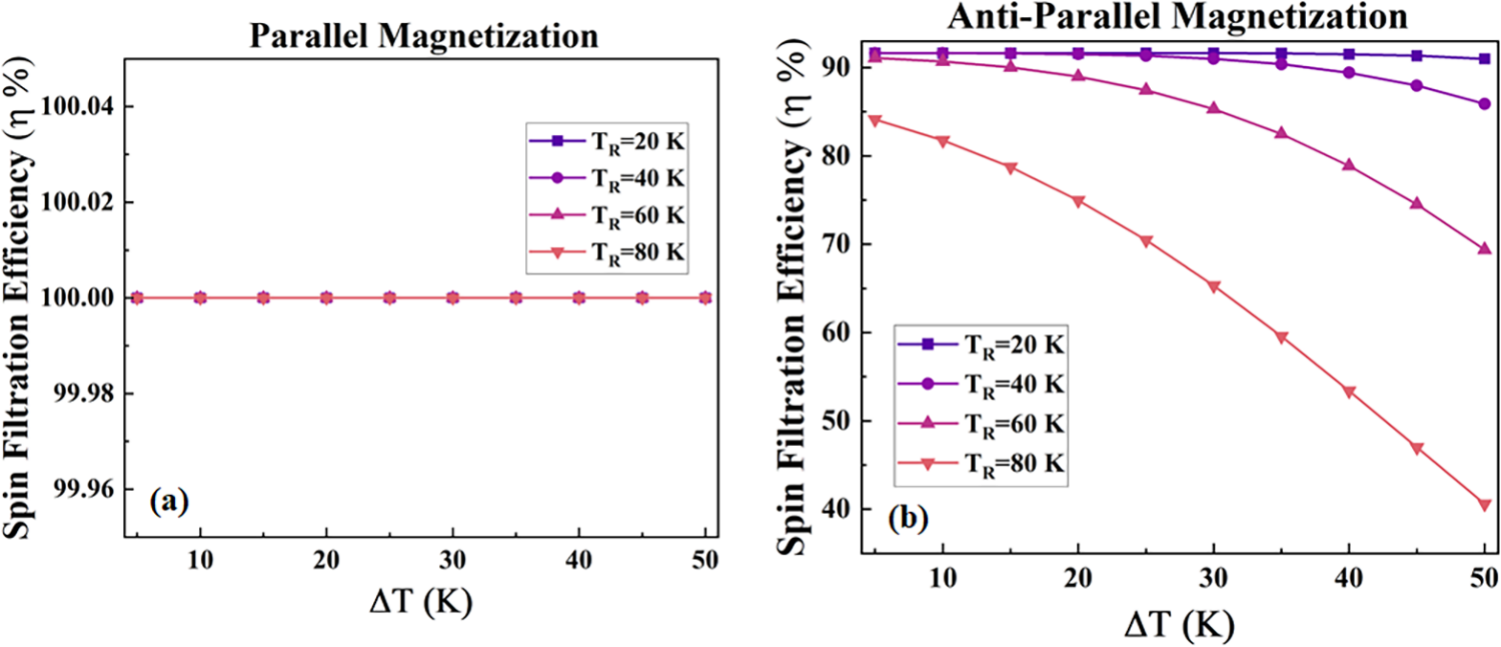
Spin filtration efficiency versus temperature difference between left and right electrodes (Δ*T*) at various right electrode temperatures (*T*_R_) for the CrO_2_/CrI_3_/2H-WTe_2_/CrO_2_ device for the configuration. (a) Parallel magnetization and (b) Antiparallel magnetization.

In addition, we have evaluated the temperature-dependent magnetoresistance with respect to Δ*T* for CrO_2_/CrI_3_/1*T*′-WTe_2_/CrO_2_ and CrO_2_/CrI_3_/2H-WTe_2_/CrO_2_ devices as shown in Figure 6a,b, respectively. The temperature-dependent magnetoresistance is computed by using the following equation:

4 $$\mathrm{MR}\left(T\right)=\frac{\left(I_{\mathrm{PM}}\left(T\right)-I_{\mathrm{APM}}\left(T\right)\right)}{I_{\mathrm{APM}}\left(T\right)}\times 100\%$$

Here, *I*_PM_ denotes the total current (up-spin (↑) + down-spin (↓)) for parallel magnetization, while *I*_APM_ denotes the total component of current for antiparallel magnetization. For the device having the 1*T*′ phase of WTe_2_ (Figure 6a), the MR value for *T*_R_ = 20 K maintains a constant value of 152.5 × 10^3^% and increases with Δ*T* for *T*_R_ = 40, 60, and 80 K. For the 2H phase of WTe_2_ (Figure 6b), the MR value for *T*_R_ = 20 K is constant at 1.45 × 10^9^% with the increase in Δ*T* but decreases for *T*_R_ = 40, 60, and 80 K to around 1.0 × 10^9^%. This behavior can be understood by considering that the 1*T*′ phase of WTe_2_ exhibits semimetallic characteristics strongly influenced by SOC. As the temperature gradient increases, more spin-polarized hot carriers contribute to transport, resulting in a larger difference in current between the parallel and antiparallel configurations, thus increasing the MR. In contrast, the 2H phase of WTe_2_ is semiconducting with a relatively larger band gap and weaker influence of SOC. For the designed device, although the MR remains high at a low value of *T*_R_, it decreases with increasing Δ*T* at higher *T*_R_. This decrease is due to the increased thermal excitation of unpolarized carriers and reduced spin filtering efficiency in 2H-WTe_2_, which causes a smaller difference in transmission between the magnetic configurations. Additionally, at elevated temperatures, magnon excitations in CrI_3_ and thermal broadening further diminish spin polarization, contributing to the decrease in MR. The nature of MR for the 1*T*′ phase of WTe_2_ is opposite, and the magnitude of the transmission is 6 orders of magnitude lower as compared to the 2H phase of WTe_2_.

**Figure 6:**
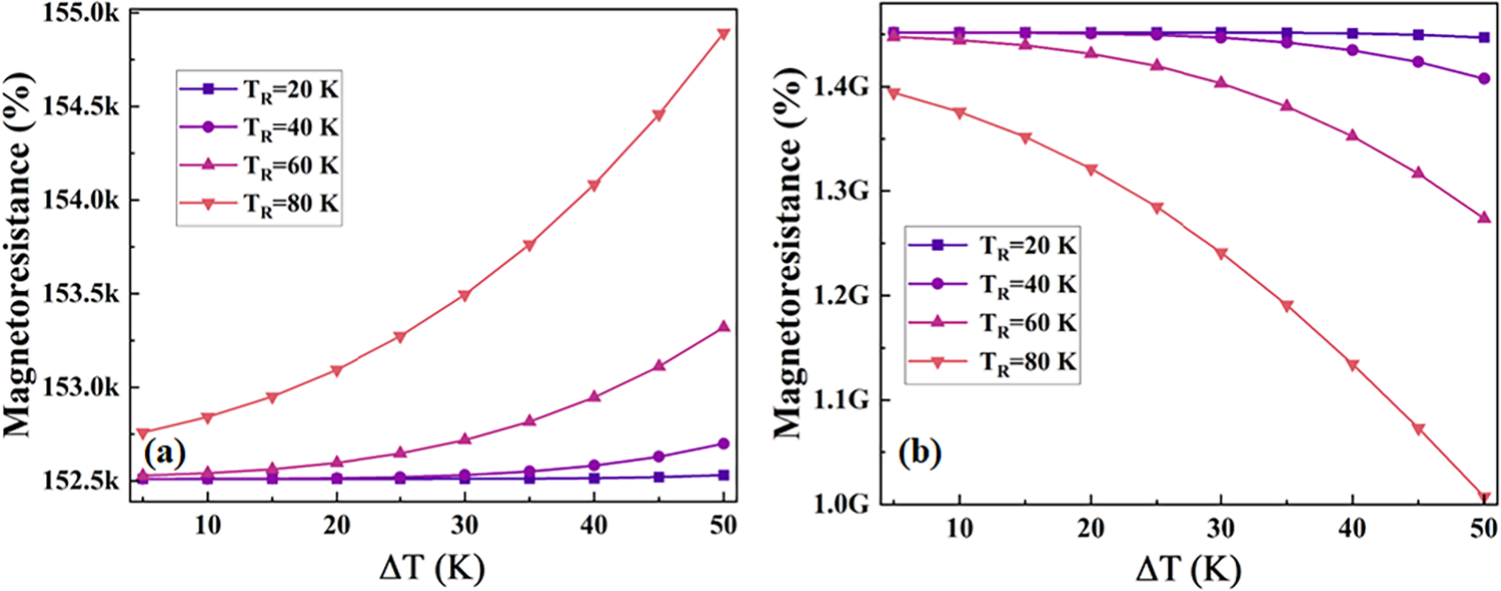
Plot for temperature-dependent magnetoresistance with respect to Δ*T* for (a) CrO_2_/CrI_3_/1*T*′-WTe_2_/CrO_2_ and, (b) CrO_2_/CrI_3_/2H-WTe_2_/CrO_2_.

## Conclusions

In conclusion, our research work describes the in-depth study of two different heterostructure devices CrI_3_/1*T*′-WTe_2_ and CrI_3_/2H-WTe_2_ using a half-metallic CrO_2_ spin-injection electrode to study the full device characteristics. By applying DFT and DFT-NEGF computational methodology, thermal spin transport at low temperatures and electronic properties were investigated for spin-caloritronic applications. The physical origin behind the spin filtration and magnetoresistance was analyzed in terms of the transmission spectrum decomposed in components of the up-spin and down-spin channels for PM and APM configurations and further associated with the density of states of the device. The performance of the devices depends strongly on the WTe_2_ allotrope, where the 6 orders of magnitude larger magnetoresistance for the 2H-WTe_2_ heterostructure is attributed to a half-metallic interface state. These unique characteristics suggest that the studied devices are promising for waste heat generation of spin currents, further improving the suitability of spintronics for green energy technology. The well-documented photoswitching properties of WTe_2_ make future exploration of optospintronics devices a promising avenue for ultrafast spintronics devices.^42,43^ Future studies can extend this work by incorporating different spin alignments within the CrI_3_ layer and explicitly modeling interfacial exchange coupling, which may lead to richer spin caloritronic behavior in such asymmetric heterostructures.

## Computational Methods

Various mesh cutoffs and k-point samplings were tested to understand the numerical convergence. For device computation, the electronic density of the bulk central region was matched with the electrodes and the central region, and then the self-consistent electronic structure of the scattering region was calculated and therefore for the complete heterostructure device to obtain the lowest possible energy configuration within the specified convergence limit. The investigation of structural properties, structure relaxation, device modeling, electronic properties, and spin-dependent transport properties is carried out within the framework of spin-polarized density functional theory (DFT), combined with the nonequilibrium Green’s function (NEGF) technique has been implemented in the Quantumwise ATK package.^44,45^ We have taken the exchange and correlation functional accompanied by the Perdew–Burke–Ernzerhof (PBE) setting to describe the spin-polarized generalized gradient approximation (SGGA). Double ζ polarized basis set (DZP) and norm-conserving pseudopotentials were used for the computation. The valence electrons of all atoms are positively dealt with the plane-wave method, and the interactions of valence electrons with ions are traced by projected augmented plane-wave (PAW) pseudopotentials.^46^ For electronic structure calculations, such as DOS, we employed PAW pseudopotentials with a plane-wave basis. On the other hand, for transport properties, we used norm-conserving pseudopotentials with a DZP basis. The norm-conserving pseudopotentials, combined with the DZP basis set, allow for a precise description of localized states while reducing computational overhead, which is particularly important when studying transport properties in the presence of complex interfaces. To optimize the structure and for calculations of electronic properties, Monkhorst–Pack *k*-point centered at γ (Γ), having grids of 7 × 7 × 1, has been taken.^47^ The Grimme (DFT-D2) functional has been used to consider the vdW interactions, and the spin–orbit coupling (SOC) is also included to calculate the electronic properties while performing the computation. SOC was included in the central scattering region of the device. However, due to current implementation limitations in QuantumATK, SOC could not be incorporated throughout the entire device setup, including the semi-infinite leads, during the transport calculations. Furthermore, the leads consist of lighter elements, for which the effects of SOC are relatively negligible, minimizing their overall impact on the transport results. All atomic coordinates, as well as the lattice constants, have been fully relaxed before performing any calculation that has an energy convergent criterion of 10^–5^ eV per unit cell, and the forces on all relaxed atoms are less than 0.01 eV Å^–1^. The cutoff energy for the density mesh was set at 75 Hartree, and initially, the electronic temperature was set at 300 K. A vacuum region of 12 Å was applied to avoid spurious interaction in the designed heterostructure. The Brillouin zone (BZ) is sampled using a 5 × 5 × 100 γ-centered Monkhorst–Pack grid.

The effect of the phonon is neglected in our computation, as we focus mainly on electronic transmission. For the structural designing of the device, a two-probe open system, which comprises mainly three parts, namely, the left electrode, central region/scattering region, and right electrode, has been set up. In our computational model, the heterostructure is coupled with the electrodes using the nonequilibrium Green’s function (NEGF) formalism. The electrodes are modeled as semi-infinite metallic contacts, and the heterostructure is connected to these electrodes at its boundaries. According to the DFT + NEGF method in the ATK code, all of the couplings with electrodes are fully included in the self-energy, which can be determined during the self-consistent calculations. The self-energy Σ_L,R_(*E*) due to the left (L) and right (R) electrodes in the DFT-NEGF formalism is given as

5 $$\Sigma_{L,R}\left(E\right)=H_{\mathrm{CL},R}^{†}g_{L,R}\left(E\right),H_{\mathrm{CL},R}$$

where *H*_CL,R_ denotes the coupling Hamiltonian between the central (scattering) region and the left or right electrode, and *g*_L,R_(*E*) is the surface Green’s function of the corresponding semi-infinite electrode. The surface Green’s function is given by

6 $$g_{L,R}\left(E\right)={\left[\left(E+i\eta \right)S_{L,R}-H_{L,R}\right]}^{-1}$$

where *H*_L,R_ is the Hamiltonian of the electrode, *S*_L,R_ is the overlap matrix, and η is the positive infinitesimal parameter ensuring causality. These self-energy terms enter the retarded Green’s function of the device region as

7 $$G\left(E\right)={\left[\left(E+i\eta \right)S_{C}-H_{C}-\Sigma_{L}\left(E\right)-\Sigma_{R}\left(E\right)\right]}^{-1}$$

where *H*_C_ and *S*_C_ are the Hamiltonian and overlap matrix of the central region, respectively. These self-energies are very essential for computing transport properties in our system, and it is a complex matrix. The real part gives rise to a shift of the energy levels, while the imaginary part gives broadening (finite lifetime) of the heterostructure energy levels. The coupling parameter (matrix) is referred to as

8 $$\Gamma_{L}\left(E\right)=i\left[\Sigma_{L}^{†}\left(E\right)-\Sigma_{L}\left(E\right)\right]$$

where Γ_L_(*E*) represents the coupling strength at energy *E*, Σ_L_(*E*) is the self-energy due to the left electrode, and Σ_L_^†^(*E*) is its Hermitian conjugate. This quantity physically accounts for the escape rate of electrons from the device region into the left electrode and plays a crucial role in determining the transmission function. A similar expression holds for the right electrode.^39,45,48,49^

## Supplementary Material



## Notes

Figure showing spin filtration efficiency for 1*T*′-WTe_2_ device configuration and projected density of states for CrI_3_/1*T*′-WTe_2_ and CrI_3_/2H-WTe_2_ (PDF)
