# Supplementary material for: Robust High Mobility Half-Metallic Interface State in CrI3/WTe2 Based Heterostructures
Source: ACS Appl Mater Interfaces. 2025 Aug 26;17(36):51448–56. doi: 10.1021/acsami.5c10764 (PMC12442000; doi:10.1021/acsami.5c10764)
Supplement: Supplementary file 1 [file am5c10764_si_001.pdf]

Supporting Information
Robust High-Mobility Half-Metallic Interface State in CrI_3/WTe_2 Based Heterostructures

Nivedita Pandey* and Oscar Grånäs

Department of Physics and Astronomy, Uppsala University, SE-751 20 Uppsala, Sweden

E-mail: nivedita.pandey@physics.uu.se

Spin Filtration Efficiency

Figure 1 depict the spin filtration efficiency with respect to ΔT for T_R=20 K, 40 K, 60 K and 80 K for the PM and APM case as shown in figure 1 (a) and 1 (b) respectively for $CrO_2/CrI_3/1T'\text{-}WTe_2/CrO_2$. The device showed a perfect 100% spin filtration efficiency for both the PM and APM case as the up spin and down spin currents are not comparable and have a difference in value. The negative value of spin filtration efficiency in the APM case, as shown in figure 1 (b), is due to the high value of the down component of the current compared to the up component of the current.

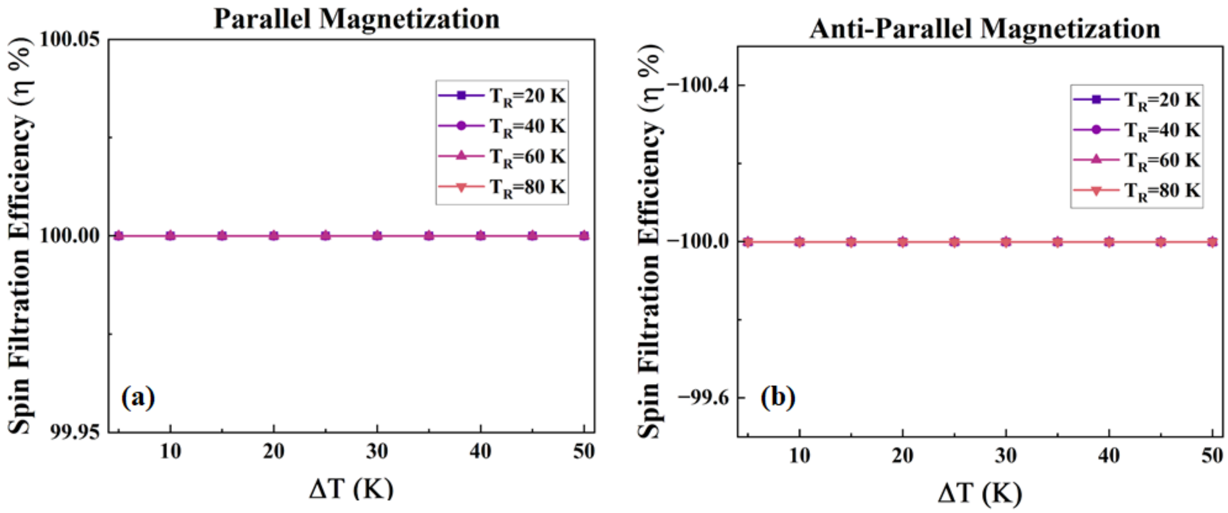

Figure 1: Spin filtration efficiency versus temperature difference between left and right electrode (ΔT) at various right electrode temperature (T_R) for $CrO_2/CrI_3/1T'\text{-}WTe_2/CrO_2$ device for the configuration (a) Parallel Magnetization, and (b) Antiparallel Magnetization.

Projected Density of States

Figure 1 (a) and Figure 1 (b) depict the PDOS for $CrI_3/1T'\text{-}WTe_2$ without SOC and with SOC, respectively. Without SOC as shown in Figure 1 (a), PDOS exhibits the states I-5p, Cr-3d, Te-5p, and W-5d near the Fermi level. The W-5d and Te-5p states contribute less as compared to the I-5p states near the Fermi level, leading to low hybridization and weak coupling in the transport channel. When SOC is included, as shown in Figure 1 (b), we observe a peak splitting of the W-5d and I-5p states, along with shifts in peak position at the Fermi level and W-5d and Te-5p contribute around the Fermi position. These changes affect the transmission spectra and carrier distribution, as the SOC modifies the available conducting channels, thereby influencing the transport characteristics. Figure 1 (c) and Figure 1 (d) depicts the PDOS for $CrI_3/2H\text{-}WTe_2$ without SOC and with SOC, respectively. Without SOC, as shown in Figure 1 (c) metallicity is induced because of Cr-3d and I-5p states in the designed heterostructure and states can be observed around the Fermi level due to decrease in bond length of the atoms upon optimization. There is induction of electrons in CrI_3 due to WTe_2 layer. The states due to W-5d and Te-5p are not present around the

Fermi level. In the case of with SOC as shown in Figure 1 (d), I-5p and some states of W-5d are present around the fermi level. Also, most of the I-5p peak and some W-5d peaks split due to inclusion of SOC because of the degeneracy in the electronic states.

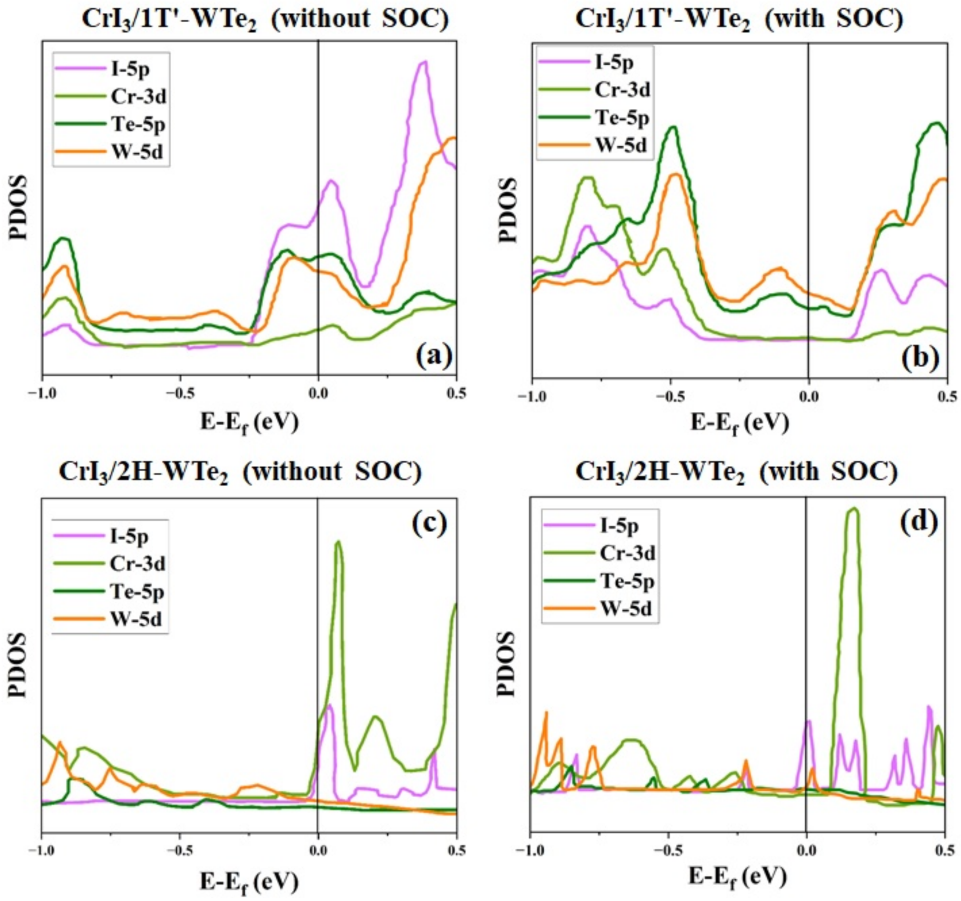

Figure 2: Depicts the projected density of states (PDOS) of $1T'$-WTe_2/CrI_3 (a) without SOC (b) with SOC, and 2H-WTe_2/CrI_3 (c) the $1T'$ phase of WTe_2, and (d) the 2H phase of WTe_2.
